# Effectiveness of screening and treatment of children with severe acute malnutrition by community health workers in Simiyu region, Tanzania: a quasi-experimental pilot study

**DOI:** 10.1038/s41598-021-81811-6

**Published:** 2021-01-27

**Authors:** Calistus Wilunda, Fortihappiness Gabinus Mumba, Giovanni Putoto, Gloria Maya, Elias Musa, Vincenza Lorusso, Chacha Magige, Germana Leyna, Fabio Manenti, Donata Dalla Riva, Bupe Abel Ntoga, Giulia Segafredo

**Affiliations:** 1 Maternal and Child Wellbeing Unit, African Population and Health Research Center, APHRC Campus, 2nd Floor, Manga Close, Off Kirawa Road, P.O. Box 10787, Nairobi, 00100 Kenya; 2 Epidemiology and Prevention Group, National Cancer Center, Tokyo, 104-0045 Japan; 3 Doctors with Africa CUAMM, Simiyu, 39101 Tanzania; 4 Doctors with Africa CUAMM, 35100 Padua, Italy; 5 Simiyu Regional Medical Officer’s Office, Simiyu, 39101 Tanzania; 6 Tanzania Food and Nutrition Centre, Dar es Salaam, 11101 Tanzania

**Keywords:** Paediatric research, Clinical trial design

## Abstract

Health system constraints hamper treatment of children with severe acute malnutrition (SAM) in Tanzania. This non-inferiority quasi-experimental study in Bariadi (intervention) and Maswa (control) districts assessed the effectiveness, coverage, and cost-effectiveness of SAM treatment by community health workers (CHWs) compared with outpatient therapeutic care (OTC). We included 154 and 210 children aged 6–59 months with SAM [mid-upper arm circumference (MUAC) < 11.5 cm] without medical complications in the control and intervention districts, respectively. The primary treatment outcome was cure (MUAC ≥ 12.5 cm). We performed costing analysis from the provider’s perspective. The probability of cure was higher in the intervention group (90.5%) than in the control group (75.3%); risk ratio (RR) 1.17; 95% CI 1.05, 1.31 and risk difference (RD) 0.13; 95% CI 0.04, 0.23. SAM treatment coverage was higher in the intervention area (80.9%) than in the control area (41.7%). The cost per child treated was US$146.50 in the intervention group and US$161.62 in the control group and that per child cured was US$161.77 and US$215.49 in the intervention and control groups, respectively. The additional costs per an additional child treated and cured were US$134.40 and US$130.92, respectively. Compared with OTC, treatment of children with uncomplicated SAM by CHWs was effective, increased treatment coverage and was cost-effective.

## Introduction

Severe acute malnutrition (SAM), defined as weight-for-height Z score < − 3 based on WHO child growth standards, directly affects 14 million children younger than five years globally^1^. However, this figure underestimates the annual SAM burden because it is based on prevalence data^2^. More than 90% of children with SAM reside in low- and middle-income countries^3^ where fragile health systems struggle to improve coverage and quality of health care, and climate change is expected to exacerbate the already food insecure situation^4^. SAM increases the risk of child mortality by more than 11-fold^3^. Therefore, there is an urgent need to develop scalable and sustainable strategies to address the problem of SAM in these settings.

In the last two decades, the model to address treatment of SAM shifted from centralized small-scale inpatient treatment to the establishment of decentralized outpatient therapeutic feeding programs through the implementation of the Community Management of Acute Malnutrition (CMAM)^5^. The key strategy of CMAM is the identification of children with SAM by community health workers (CHWs) or volunteers, referral of such children to health facilities for assessment by professional health workers, and outpatient or inpatient treatment of the undernourished children. This strategy was designed to improve treatment outcomes and coverage through early detection and early treatment initiation of malnourished children. However, a review of 44 CMAM programs in 21 countries showed that most of them did not reach the minimum coverage standards set by the Sphere project (i.e. 50%, 70% and 90% for rural, urban, and camp settings, respectively)^6^. The most important barrier to access was lack of engagement with beneficiary communities^7^, suggesting that the current service delivery model is unable to provide the level of access required by beneficiary communities. Thus, innovative delivery strategies—especially community-based delivery platforms—for the scale-up of SAM services are urgently needed^8^.

A national nutrition prevalence survey in 2014 found that 4.5% of children in Tanzania had acute malnutrition, with 1.2% having SAM^9^. The country is currently implementing CMAM with community health workers (CHWs) playing a key role in this, although they are not officially integrated in the national health system because they work as volunteers without official remuneration and a scheme of service. Health facilities with units for treatment of malnutrition are often located in towns, creating problems in access to care due to long distances from households to health facilities and between health facilities, weak links between the community and facilities in referring malnourished children, and indirect costs. There are also health system constraints such as staff shortages, limited training and supervision, and lack of necessary equipment and ready to use foods that hamper the management of acute malnutrition^10^. The result is a low coverage of SAM services, a high relapse rate, and a high case fatality rate that is above the acceptable range of 5–10% ^10^.

Studies from Africa^11,12^ and Asia^13^ show that malnourished children with no complications treated at home by CHWs have a higher cure rate and lower default rate than those treated in health facilities. There is also evidence that CHWs can deliver good quality care^14^ and using them to treat SAM is cost-effective^15^. However, there is no local evidence from Tanzania where the role of CHWs is limited to screening and referring malnourished children to health facilities for treatment. The current national guidelines for Integrated Management of Acute Malnutrition stipulate timely detection of SAM in the community and provision of treatment for those without medical complications with ready-to-use therapeutic foods (RUTF) or other nutrient-dense foods through outpatient therapeutic care (OTC)^10^.

To improve access to treatment of SAM, Doctors with Africa *Collegio Universitario Aspiranti Medici Missionari* (CUAMM), an Italian non-governmental organisation operating in Tanzania since 1968, piloted a model to screen and treat children with SAM without complications using CHWs. This pilot study was nested within a large four-year project named “The Next Generation Programme—Integrated Promotion of Nutrition, Growth and Development” in Simiyu and Ruvuma regions funded by Children’s Investment Fund Foundation. This study aimed to assess the effectiveness and cost-effectiveness of treatment of SAM by CHWs, and the effect of this intervention on SAM treatment coverage.

## Methods

### Study setting

This study was conducted in Simiyu Region, northern Tanzania, where in 2018, 4.6% of children were acutely malnourished with 0.5% being severely acutely malnourished^16^. According to the 2012 census, Simiyu Region had a population of about 1.6 million inhabitants and was divided into five districts. In consultation with the local health authorities, three rural wards (Sakwe, Ihusi and Mwadobana) in Bariadi District and three rural wards (Malampaka, Busilili, Shishiyu) in Maswa District were selected purposively as intervention and control areas, respectively. In selecting the intervention and control areas, the following factors were considered: study logistics, distance between the two areas to minimize contamination, comparability between the two areas in terms of the population size, expected number of SAM cases, and health infrastructure—number of CHWs working on the Next Generation Programme, number of health facilities, and distance between wards and SAM treatment centres. The intervention wards had a population of about 45,200 people distributed in 11 villages and served by 13 CHWs, three dispensaries and one health centre. The control wards had a population of about 35,800 people distributed in nine villages and served by 11 CHWs, three dispensaries and one health centre.

### Study design and participants

This is a parallel two-arm non-inferiority quasi-experimental pilot study. All children aged 6–59 months with SAM and without medical complications were eligible for inclusion if their primary caretakers provided consent. Eligible children were recruited in the community by CHWs in the intervention wards or by formal health workers in health facilities in the control wards. Only children with good appetite, without severe oedema and no underlying medical condition and/or complications were eligible for enrolment in the study. In the intervention area, CHWs screened children for SAM by measuring their mid-upper arm circumference (MUAC) and those with MUAC < 11.5 cm or mild/moderate oedema were classified as having SAM and treated at home using RUTF, with the dosage based on a child’s body weight. CHWs followed up enrolled children through weekly home visits to replenish their RUTF and to monitor their progress by assessing their weight, MUAC, and medical symptoms.

In the control wards, CHWs screened and referred malnourished children to nearby health facilities for treatment by health workers according to the standard national guidelines^10^. Caretakers could also take their children directly to health facilities. Health workers enrolled children in the study using criteria similar to that used in the intervention district. Supplemental Fig. S1 shows the flow chart used in this study (adapted from national guidelines^10^) for screening and management of children with acute malnutrition by CHWs. All enrolled children were followed up—either by CHWs in the intervention wards or health care workers through OTC clinics in the control wards—until they exited the study after experiencing one of the study outcomes.

Prior to the intervention, CHWs and their supervisors (who included the program staff and health facility staff who usually supervise CHWs in their catchment areas) were adequately trained to screen and manage children with SAM. The training, which covered both theory and practice, was delivered by nutritionists from the Tanzania Food and Nutrition Centre and aimed to impart knowledge and skills in management of SAM among children younger than five years old at the community level. CHWs and their supervisors from the intervention area received further training on home treatment of children with SAM without medical complications.

### Study outcomes and data collection

We defined study outcomes in a standard way in both the intervention and control groups. The primary study outcome was cure from SAM, defined as MUAC ≥ 12.5 cm. The secondary study outcomes were default, defined as absence on three consecutive visits; non-response, defined as failure to attain discharge criteria after three months on treatment; transfer to inpatient therapeutic care (ITC); or death. The criteria for ITC transfer were loss of appetite, development of medical complications, development of oedema, weight loss or static weight on three consecutive visits, and request by the caregiver. Other secondary outcomes were length of stay, defined as the number of days from treatment initiation to recovery and average weight gain, defined as weight change (g per kg per day) from treatment initiation to recovery. Baseline maternal and child’s sociodemographic data (child’s sex and age; mother’s vital status, age, education, and household wealth variables) and child’s physical assessment and health status data (MUAC, weight, exposure to HIV, type of admission, and presenting symptoms) were collected at enrolment. Child’s MUAC, weight and the amount of RUTF dispensed were recorded at each weekly visit. All collected data were recorded in case report forms contained in an enrolment and follow-up register. Children were enrolled into the study from August 2018 to December 2019 in the intervention group and from August 2018 to February 2020 in the control group. Follow-up ended on 26 March 2020.

We obtained data to estimate coverage from SAM registers in health facilities in the control wards and from CHWs in the intervention wards. We also reviewed SAM registers at three health facilities (Maswa, Somanda and Songambele) offering ITC in the study districts and counted all children from the study wards who were treated in these health facilities. The main source of cost data was the accounting records of Doctors with Africa CUAMM (the implementing agency). We collected additional cost data on human resources (salaries and time allocation), capital and consumables using a questionnaire administered to health facility staff in the control areas.

### Sample size

We estimated the minimum required sample size of 258 (129 per group) assuming that treatment of children with SAM by CHWs was non-inferior compared to treatment of children with SAM in health facilities, an overall proportion of cured children in both arms of 88% (pi = 0.88), a non-inferiority margin of 10% (delta = 0.1), a power of 80%, and a one-sided alpha of 0.025. We used the *ssi* module in Stata (College Station, TX, USA) to calculate the sample size.

### Data management and analysis

#### Effectiveness analysis

Data were entered in EpiData in duplicate, validated and exported to Stata 15 for cleaning and analysis, which was performed based on the intention-to-treat principle. Characteristics of participants were summarized using descriptive statistics and differences between intervention and control groups were compared using independent samples t-tests (for continuous variables) or chi-squared tests (for categorical variables). Six children in the control group had missing outcome data because follow-up ended before we could ascertain their outcomes, thus, we performed both complete-case analysis and analysis after multiple imputation to account for the missing data. We used multiple imputation with chained equations with 20 iterations based on all maternal and child characteristics listed in Table 1. We calculated risk ratios (relative effects) and risk differences (absolute effects) with 95% CIs for cure and default using Poisson regression models with robust error variances^17^. We assessed the effect of the intervention on length of stay and weight gain using linear regression to obtain mean differences with 95% CIs. We adjusted the models for variables that showed some imbalance (P < 0.1) between control and invention groups. Estimates across imputed datasets were automatically combined using Rubin’s rules^18^. To evaluate non-inferiority of the intervention compared to the usual care, we compared the lower bound of the 95% CI for the effect of the intervention on cure with the pre-specified non-inferiority margin (− 10%). We did not assess the effect of the intervention on death, transfer and non-response to treatment because of a small number of observations.

**Table 1:** Characteristics of study participants at recruitment.

Characteristics	Control (N = 154)	Intervention (N = 210)	P-value
**Child’s sex**			0.622
Male	70 (45.5)	90 (42.9)	
Female	84 (54.5)	120 (57.1)	
**Child’s age, months**	15.1 ± 7.8	15.0 ± 8.5	0.983
**MUAC, cm**	11.0 ± 0.7	11.1 ± 0.5	0.156
**Weight, kg**	6.7 ± 1.3	6.7 ± 1.2	0.702
**Exposed to HIV**			< 0.001
No	116 (75.3)	181 (86.2)	
Yes	28 (18.2)	10 (4.8)	
Not known	10 (6.5)	19 (9.1)	
**Mother alive**			0.146
No	7 (4.6)	4 (1.9)	
Yes	147 (95.5)	206 (98.1)	
**Caretaker’s age, years**	29.0 ± 9.8	27.5 ± 8.5	0.177
**Mother’s education**			0.382
None	46 (30.5)	77 (37.8)	
Primary	101 (66.9)	123 (60.3)	
Secondary +	4 (3.7)	4 (2.0)	
Missing	3	6	
**Household wealth index**^**a**^			< 0.001
Lowest	53 (34.4)	26 (12.4)	
Second	20 (13.0)	47 (22.4)	
Middle	18 (11.7)	57 (27.1)	
Fourth	22 (14.3)	51 (24.3)	
Highest	41 (26.6)	29 (13.8)	
**Type of admission**			0.431
New admission	144 (93.5)	198 (94.3)	
Re-admission	4 (2.6)	8 (3.8)	
Transfer from ITC	6 (3.9)	4 (1.9)	
**Poor appetite**	42 (27.3)	44 (30.0)	0.161
**Cough**	35 (22.7)	38 (18.1)	0.276
**Vomit**	13 (8.4)	14 (6.7)	0.523
**Diarrhoea**	28 (18.2)	29 (13.8)	0.257
**Fever**	39 (25.3)	34 (16.2)	0.032
**Skin abnormality**	12 (7.8)	13 (6.2)	0.551

Data are presented as n (%) for categorical variables or Mean ± SD for continuous variables.

^a^Derived using principal components analysis of household assets, access to utilities and type of housing material.

Because the results of both multiple imputation and complete-case analysis may be biased given that only the control group had children with missing outcome data, we performed sensitivity analysis (using the same approach as above) after excluding 31 children enrolled in the study during the same period as the children with missing data (i.e. after 28th December 2019). In other words, we restricted the analysis to only those children we could have potentially followed up for the maximum follow-up period of three months.

#### Coverage analysis

The effect of the intervention on coverage, defined as the proportion of the children with SAM being reached with treatment in the intervention and control wards, was assessed using data on the number of children treated over a 12-month period from September 2018 to August 2019. We estimated coverage using an indirect method by dividing the number of children aged 6–59 months with SAM who received treatment (including ITC) by the expected number of children aged 6–59 months with SAM over the reference period (the annual SAM burden). Where $$Annual SAM burden =number of children 6-59 months*SAM prevalence*(1+K)$$; K being the incidence correction factor, whose value was assumed to be 4.82 based on a meta-analysis of studies from three West African countries^19^ (similar data for Tanzania/East Africa are not available). We used a SAM prevalence 0.5% for Simiyu Region based on the National Nutrition Survey 2018^16^. To estimate the effect of the intervention on coverage, we calculated both relative and absolute changes in coverage.

#### Cost-effectives analysis

We performed cost-effectives analysis from the provider’s perspective. The time horizon was 1 year: from September 2018 to August 2019. We calculated costs using the activity-based costing method by identifying the activities of the project, determining the cost of each activity and calculating the overall and unit costs. Cost analysis focused on treatment of children with SAM without complications at the ward level. Thus, we did not consider ITC costs. We included costs related to sensitization and mobilization, training of CHWs and their supervisors (transportation of trainers, training hall and meals, and per diems), supervision and monitoring (fuel costs and per diems), personnel costs (staff salaries and benefits, and incentives for CHWs and supervisors), consumables (RUTF purchase and transportation, photocopying and binding, drugs, bicycle maintenance and spare parts) and capital costs (weighing scales, thermometers, MUAC tapes, clinic furniture, and room rent). The quantity of RUTF dispensed was as reported in the child enrolment and follow-up register (from admission to discharge). Personnel costs were adjusted for time spent on the project. All costs were expressed in 2019 US dollars (1 TZ = 0.0004 US$). Capital items (any item that can be used for more than one year), were annualized using a 3% interest rate and corresponding useful life. The same strategy was used in estimating the cost of sensitization/mobilization and trainings. We computed the unit cost i.e. cost per child treated and cost per child cured. In addition, we calculated incremental cost-effectiveness ratio (ICER) by dividing the difference in costs incurred in the intervention and control areas by the difference in the number of children treated or cured in the intervention and control areas (i.e. C_1_ − C_0_/E_1_ − E_0_). We analysed the data using Microsoft Excel.

### Ethical considerations

The National Health Research Ethics Committee at the National Institute of Medical Research, Tanzania (NIMR/HQ/R.8a/Vol.IX/2532) approved the study protocol. This study complied with the ethical standards set by the National Health Research Ethics Committee on research regarding human subjects and with the Helsinki Declaration. Written informed consent was obtained from caretakers of all participating children before recruitment. This study was registered in the Pan African Clinical Trial Registry (Trial number PACTR201901856648139) on 21/12/2018.

### Disclaimer

Views expressed in this study are solely those of the authors and do not necessarily represent the official position of Doctors with Africa CUAMM or Children’s Investment Fund Foundation.

## Results

Three hundred and sixty four children (154 in the control group and 210 in the intervention group) were recruited in the study. In the intervention group, all recruited children were followed up until they exited the study according to the protocol. However, in the control group, the study ended before outcomes for six children (3.9%) could be ascertained. Overall, children in the intervention group were followed up for a median of 6 weeks [interquartile range (IQR) 5–8] while those in the control group were followed up for a median of 4 weeks (IQR 3–5). Table 1 shows the characteristics of the study participants at recruitment by study arm. More than half of the children (54.5% in the control group and 57.1% in the intervention group) were female. Overall, children in this study had a mean MUAC of 11 cm, a mean weight of 6.7 kg and more than 93% were new admissions. There was no statistically significant difference between the control and intervention groups with respect to most of the children’s and maternal characteristics including child’s age; sex; MUAC; and weight, mother’s age; vital status; and education, and type of admission. Significant differences were observed for HIV exposure status, wealth index and fever. Children in the control group were more likely to have been exposed to HIV and to have fever than those in the intervention group. Most of the children in the control group were in the lowest (34.4%) and highest (26.6%) wealth quintiles. On the contrary, in the intervention group, the middle wealth quintile had the highest the proportion of children (27.1%).

### Treatment outcomes

Treatment outcomes were similar in the control group with or without imputation of missing outcomes (Table 2). Cure rate was higher in the intervention group (90.5%) than in the control group (75.7%) while defaulter rate was higher in the control group (21.0%) than in the intervention group (6.5%). Only a small number of children were transferred to ITC, died, or did not respond to treatment. The two deaths reported in the intervention area occurred while the children were under the care of professional health workers after being referred by CHWs because of illness. Length of stay was slightly higher in the intervention group (34.3 days) than in the control group (30.8 days) but children in both groups had similar average weight gain (6 g/kg/day).

**Table 2:** Treatment outcomes.

Outcome	Control		Intervention (N = 210)
	Missing outcomes excluded (N = 148)	Missing outcomes imputed (N = 154)	
	n (%)	n (%)	n (%)
Cured	112 (75.7)	116 (75.3)	190 (90.5)
Defaulted	31 (21.0)	32 (20.8)	13 (6.5)
Transferred to ITC	3 (2.0)	3 (2.0)	3 (1.4)
Died	1(0.7)	1 (0.7)	2 (1.0)
No response	1 (0.7)	2 (1.3)	2 (1.0)
Length of stay (days)^a^	30.8 ± 18.6	31.0 ± 18.4	34.3 ± 18.2
Average weight gain (g/kg/day)^a^	6.4 ± 4.3	6.3 ± 4.3	6.3 ± 3.9

^a^Applies to cured children only. Data presented as Mean ± SD.

### Effect of the intervention on treatment outcomes

Table 3 shows that after adjustment for wealth index, exposure to HIV, and fever at baseline, children in the intervention group were more likely to be cured than those in the control group (RR 1.17, 95% CI 1.05, 1.31 and RD 0.13; 95% CI 0.04, 0.23). In line with this, the probability of defaulting was significantly lower in the intervention group than in the control group (RR 0.29, 95% CI 0.15, 0.56 and RD − 0.16; 95% CI − 0.23, − 0.08). Accounting for missing outcomes through imputation did not materially change the effect estimates. The intervention did not have a statistically significant effect on length of stay or weight gain before or after adjustment for potential confounders (Table 3).

**Table 3:** Effect of the intervention on treatment outcomes.

Outcome	Unadjusted				Adjusted^1^			
	RR (95% CI)	P value	RD or MD (95% CI)	P value	RR (95% CI)	P value	RD or MD (95% CI)	P value
**Missing outcomes excluded**								
Cure (N = 358)	1.20 (1.08, 1.32)	0.001	0.15* (0.07, 0.23)	< 0.001	1.17 (1.05, 1.31)	0.006	0.13* (0.04, 0.23)	0.005
Default (N = 358)	0.30 (0.16, 0.55)	< 0.001	− 0.15* (− 0.22, − 0.07)	< 0.001	0.29 (0.15, 0.56)	< 0.001	− 0.16* (− 0.23, − 0.08)	< 0.001
Length of stay (N = 302)	–		3.49^†^ (− 0.81, 7.79)	0.112	–		3.70^†^ (− 0.46, 7.86)	0.081
Average weight gain (N = 302)	–		− 0.05^†^ (− 1.02, 0.91)	0.911	–		− 0.65^†^ (− 1.72, 0.42)	0.234
**Missing outcomes imputed**								
Cure (N = 364)	1.20 (1.09, 1.33)	< 0.001	0.15* (0.07, 0.23)	< 0.001	1.18 (1.05, 1.32)	0.004	0.14* (0.05, 0.23)	0.003
Default (N = 364)	0.30 (0.16, 0.55)	< 0.001	− 0.15* (− 0.22, − 0.07)	< 0.001	0.30 (0.16, 0.58)	< 0.001	− 0.15* (− 0.23, − 0.08)	< 0.001
Length of stay (N = 306)	–		3.32^†^ (− 0.93, 7.56)	0.125	–		4.02^†^ (− 0.15, 8.20)	0.059
Average weight gain (N = 306)	–		− 0.02^†^ (− 0.97, 0.94)	0.974	–		− 0.49 ^†^ (− 1.55, 0.57)	0.366

RR: Risk ratio; RD: Risk difference; MD: Mean Difference.

^1^Adjusted for wealth index, exposure to HIV, and fever.

* Risk difference.

^†^Mean difference.

The effects of the intervention on transfer, death and no response were not evaluated because of the small number of cases.

**Figure 1:**
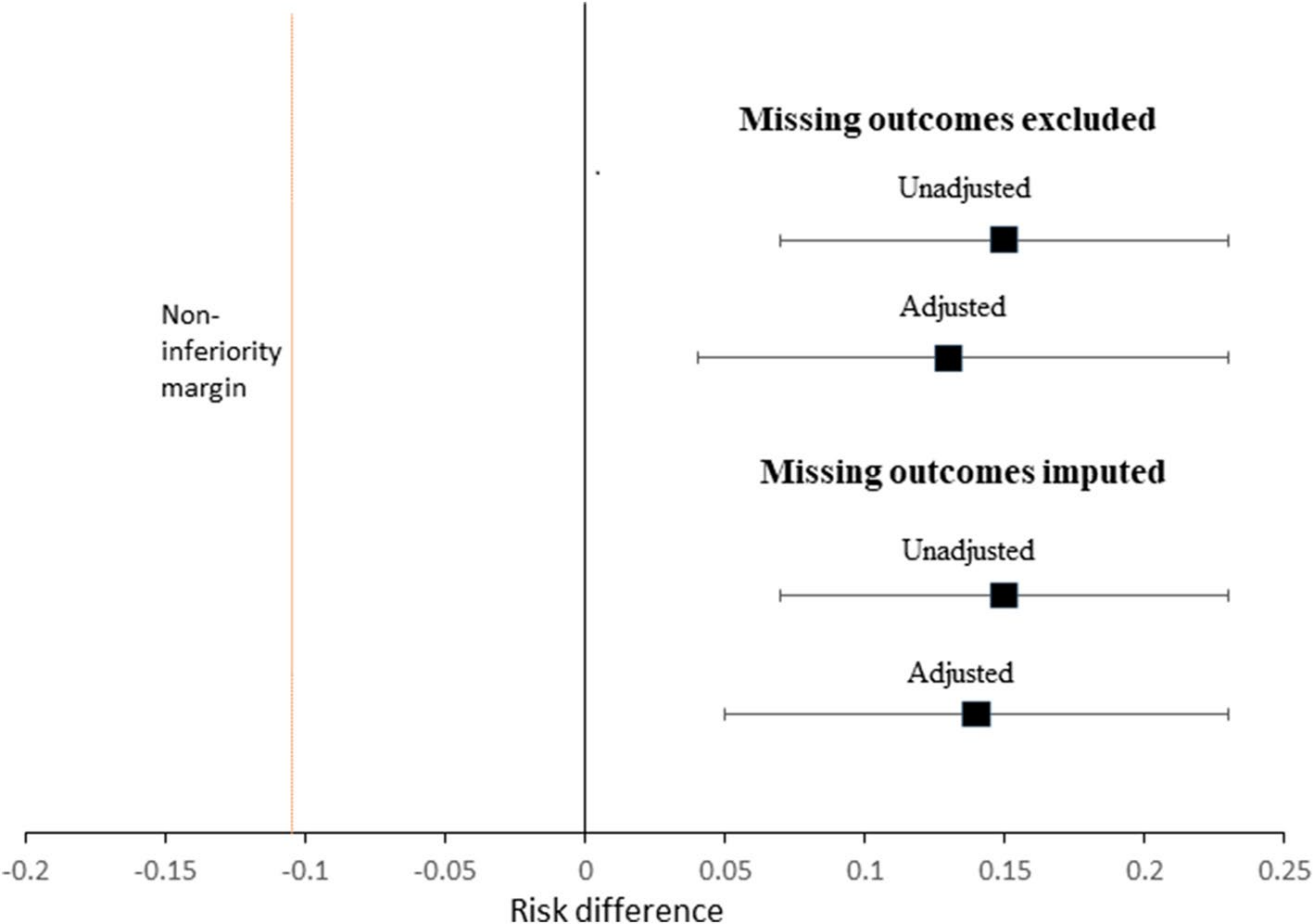
Assessment of non-inferiority based on cure rate. The risk difference (RD) is adjusted for wealth index, exposure to HIV, and fever at baseline. The error bars represent 95% CIs around the point estimate (black square).

Based on the pre-specified non-inferiority margin of 10% (RD − 0.1), the intervention was non-inferior compared to the usual care: the lower bound of the 95% CI for the RD (0.5%) is above the non-inferiority margin (Fig. 1). Because the 95% CI excludes the null value, the intervention was also superior compared to the usual care.

Sensitivity analysis after excluding all children enrolled in the study during the same period as the children with missing data did not materially change our results (Supplemental Tables S1–S3). In the control group, cure rate was 76.1% while the default rate 20.5%. There was no major change in the effect estimates (RR for cure was 1.15; 95% CI 1.02, 1.29 while the RD was 0.12; 95% CI 0.02, 0.21), demonstrating the robustness of our results.

### Coverage

Table 4 shows that SAM treatment coverage was higher in the intervention area (80.9%) than in the control area (41.7%); RD 39.2 (95% CI 30.9, 47.6) and RR 1.94 (95% CI 1.63, 2.31).

**Table 4:** Estimation of coverage of severe acute malnutrition treatment services.

	Control	Intervention
No. of children aged 0–59 months^a^	9461	11,195
No. of children aged 6–59 months^b^	8465	10,016
SAM prevalence^c^	0.005	0.005
Incidence correction factor (K)^d^	4.82	4.82
Expected annual SAM cases in children aged 6–59 months^e^	204	241
No. of children treated in 1 year (Sept 2018–Aug 2019)	85	195
SAM treatment coverage	41. 7%	80.9%
Relative change (intervention/control)	1.9 (95% CI 1.6, 2.3)	
Absolute change (intervention-control)	39.2% (95% CI 30.9%-47.6%)	

^a^Based of the 2015 projected census data for 2018/2019.

^b^89.47% of the < 5 population based on the 2015/16 DHS data.

^c^National Nutrition survey 2018, Simiyu Region.

^d^Based on meta-analysis of studies from West Africa^19^.

^e^No. of children aged 6–59 months × SAM prevalence × (1 + K). The 95% CIs were calculated using a calculator based on published formulae.

### Cost-effectiveness

Table 5 shows that the total cost was higher in the intervention group (US$ 26,369.15) than in the control group (US$ 12,929.35). Consumables, mainly the cost of RUTF and its shipment, accounted for the highest share of the total cost in both the intervention group (57.0%) and the control group (50.2%). The shares of training and supervision/monitoring costs were higher in the intervention group than in the control group while the share of human resource cost was higher in the control group than in the intervention group.

**Table 5:** Cost analysis.

	Control group		Intervention group	
	Cost (US$)	% Total cost	Cost (US$)	% Total cost
**Total cost**				
(1) Sensitization and mobilization	0	0.0	422.69	1.6
(2) Training	763.67	5.9	2590.18	9.8
(3) Monitoring and supervision	560.00	4.3	3062.86	11.6
(4) Consumables	6,492.05	50.2	15,018.51	57.0
(5) Human resources	4,555.95	35.2	4868.73	18.5
(6) Capital costs	557.68	4.3	406.19	1.5
Total cost	12,929.35	100.0	26,369.15	100.0

Unit cost (US$)				
Number of children treated	80		180	
Number of children cured	60		163	
Cost per child treated	161.62		146.50	
Cost per child cured	215.49		161.77	

Incremental cost-effectiveness ratio	Treated	Cured		
C1-C2	13,439.80	13,439.80		
E1-E2	100	103		
ICER (C1-C2/E1-E2)	134.40	130.92		

*C* cost, *E* effect, *ICER* Incremental Cost Effectiveness Ratio.

The cost per child treated was lower in the intervention group (US$ 146.50) than in the control group (US$ 161.62). Similarly, the cost per child cured was lower in the intervention group (US$ 161.77) than in the control group (US$ 215.49). The additional cost per an additional child treated (ICER) was US$134.40 while that per an additional child cured was US$130.92.

## Discussion

This study showed that using CHWs to treat children with uncomplicated SAM was superior compared to the standard OTC model. Children treated by CHWs attained a higher cure rate and were less likely to default compared with those treated in health facilities. Moreover, the intervention increased coverage of SAM treatment services. Given Tanzania’s per capita GDP of $1105 in 2019, the ICERs of $134.4 per treated child and $130.92 per cured child suggest that the intervention is cost-effective in this setting.

Our findings are consistent with the accumulating evidence on the effectiveness and cost-effectiveness of treatment of SAM by CHWs. A recent review that included 12 peer-reviewed articles and 6 grey literature from Africa and Asia on management of uncomplicated SAM by CHWs showed that CHWs could identify and treat uncomplicated cases of SAM, achieving cure rates above the minimum standards and reducing default rates^20^. Despite the discrepancies in treatment protocols used (in terms of admission criteria, treatment and discharge criteria), the review found cure rates of above 75% in the intervention group in eight out of nine studies. Cure rates of > 90% were reported in Angola^21^, Bangladesh^13^, Malawi^22^, and Mali^12^. Default rate was < 8% across all the studies, ranging from 3.6% in Malawi to 7.5% in Bangladesh. The review further found that only three studies (from Angola, Mali and Bangladesh) reported coverage; the post intervention coverage ranged from 82.1% in Angola to 89% in Bangladesh. Although coverage in these studies was assessed directly using the semi‐quantitative evaluation of access and coverage (SQUEAC)/simplified lot quality assurance sampling evaluation of access and coverage methods, these results are consistent with what we found in our study based on indirect assessment using service use data. A SQUEAC assessment conducted in Simiyu Region in 2018 found a SAM treatment coverage of 39.9% (95% CI 29.2–52.0%) [unpublished report], which is similar to our estimated coverage of 41.7% in the control area. It is worth noting that, in the intervention area, the treatment outcomes and coverage were above the Sphere standards (i.e., > 75% recovered, < 15% defaulted, < 10% died, and > 50% SAM treatment coverage in rural areas)^6^. In the control area, only the recovery rate met the Sphere standards.

Assessment of SAM treatment via CHWs was found to be cost-effective^11^. Two studies have assessed the cost‐effectiveness from the community perspective. In Bangladesh, in the group treated by CHWs, the costs per child treated and child recovered were $165 and US$180, respectively^15^. In Malawi, it cost US$244 per child treated and US$259 per child recovered in the intervention area. The respective costs in the control group were US$442 and US$501. In an evaluation of a CMAM program in Ethiopia where costing was assessed from the provider’s perspective, the average cost per treated child was US$110, ranging from US$90 to US$152^23^, which is close to our finding. In line with other studies, our study shows that the cost of RUTF accounts for the highest share of the total cost. Further analysis showed that shipment cost was the main driver of the cost of RUTF. This was mainly because RUTF was imported from Europe. Thus, local manufacture of RUTF could significantly lower the cost of SAM treatment. Overall, consistent with other studies, the intervention was cost-effective.

To our knowledge, this is the first study to assess the effectiveness of SAM treatment by CHWs in Tanzania. This was a pilot study with some limitations worth highlighting. First, due to budgetary constraints, we did not assess the quality of care by CHWs. Despite this, studies have shown that CHWs generally provide good quality care in SAM management^20^. Moreover, the good treatment outcomes observed in the intervention area indicate that the quality of care provided by CHWs was good. Secondly, we did not assess the perceptions of different stakeholders, including CHWs and beneficiaries, towards this new SAM treatment model. Such information can be useful in designing a bigger study or in scaling up the intervention. Thirdly, this being a non-randomised study, there is a possibility of confounding. In particular, there was a higher proportion of children exposed to HIV in the control group than in the intervention group. This is likely to yield better treatment outcomes in the intervention group. Moreover, there was an imbalance in the socio-economic status of children between the study groups, with a seemingly greater socio-economic disparity in the control group than in the intervention group. Nonetheless, adjustment for these factors did not change the effect estimates. Although a randomized controlled trial (RCT) would be ideal in addressing the limitations of this quasi-experimental study, the difficulty of conducting an RCT and the required financial resources means this type of study design may provide the best evidence about the intervention in this resource-limited setting. We estimated coverage indirectly based on utilisation data. Nonetheless, our estimated coverage in the control area was similar to that of Simiyu Region obtained through a direct method. Finally, we performed costing analysis at the ward level and did not include costs incurred in treating children referred to higher levels. However, given that the number of children referred to ITC was low and equal between the groups, this is unlikely to influence the ICER.

In conclusion, treatment of children with SAM without complications by CHWs was more effective and non-inferior compared with the usual care. The intervention led to higher coverage of SAM treatment and was cost-effective. The results from this study together with the accumulating evidence from elsewhere form a strong case for promoting the use of CHWs to manage children with SAM at home in Tanzania and other resource limited settings. This is particularly relevant in the context of the ongoing COVID-19 pandemic where limiting visits to the health facilities to the minimum needed is highly desirable.

## Supplementary Information

Supplementary Information.
