# Supplementary material for: Effectiveness of screening and treatment of children with severe acute malnutrition by community health workers in Simiyu region, Tanzania: a quasi-experimental pilot study
Source: Sci Rep. 2021 Jan 27;11:2342. doi: 10.1038/s41598-021-81811-6 (PMC7840757; doi:10.1038/s41598-021-81811-6)
Supplement: Supplementary file 1 — Supplementary Information. [file 41598_2021_81811_MOESM1_ESM.docx]

**Effectiveness of Screening and Treatment of Children with Severe Acute Malnutrition by Community Health Workers in Simiyu Region, Tanzania: A Quasi-Experimental Pilot Study**

Calistus Wilunda^1,2^, Fortihappiness Gabinus Mumba^3^, Giovanni Putoto^4^, Gloria Maya^3^, Elias Musa^3^, Vincenza Lorusso^3^, Chacha Magige^5^, Germana Leyna^6^, Fabio Manenti^4^, Donata Dalla Riva^4^, Bupe Abel Ntoga^6^, Giulia Segafredo^3^

^1^ Maternal and Child Wellbeing Unit, African Population and Health Research Center, Nairobi, 00100, Kenya

^2^Epidemiology and Prevention Group, National Cancer Center, Tokyo, 104-0045, Japan

^3^ Doctors with Africa CUAMM, Simiyu, 39101, Tanzania

^4^ Doctors with Africa CUAMM, Padua, 35100, Italy

^5^ Simiyu Regional Medical Officer's Office, Simiyu, 39101, Tanzania

^6^ Tanzania Food and Nutrition Centre, Dar es Salaam, 11101, Tanzania

**Child with acute malnutrition**

- MUAC <11.5 cm
- Bilateral pitting oedema

<6 months

6-59 months

Oedema +++

Oedema 0, +, ++

Check for medical complications and perform an appetite test

Medical complications

or

Fails appetite test

No medical complications and

Passes appetite test

Treat at home with RUTF

Antibiotic treatment

Deworming for children aged >1 year

Refer to the health facility

**Supplemental Figure 1: An approach for screening and management of acute malnutrition by community health workers.**

**Supplemental Tables**

| **Characteristics** | | **Control (N=117)** | **Intervention (N=210)** | **P-Value** |
| --- | --- | --- | --- | --- |
| Child’s sex | |  |  | 0.565 |
|  | Male | 54 (46.2) | 90 (42.9) |  |
|  | Female | 63 (53.9) | 120 (57.1) |  |
| Child’s age, months | | 14.4±6.5 | 15.0±8.5 | 0.533 |
| MUAC, cm | | 11.0±0.6 | 11.1±0.5 | 0.670 |
| Weight, kg | | 6.7±1.3 | 6.7±1.2 | 0.935 |
| Exposed to HIV | |  |  | 0.001 |
|  | No | 90 (76.9) | 181 (86.2) |  |
|  | Yes | 20 (17.1) | 10 (4.8) |  |
|  | Not known | 7 (6.0) | 19 (9.1) |  |
| Mother alive | | 112 (95.7) | 206 (98.1) | 0.209 |
| Caretaker’s age, years | | 29.7±10.6 | 27.5±8.5 | 0.094 |
| Mothers education | |  |  | 0.469 |
|  | None | 36 (31.0) | 77 (37.8) |  |
|  | Primary | 77 (66.4) | 123 (60.3) |  |
|  | Secondary+ | 3 (2.6) | 4 (2.0) |  |
|  | Missing | 1 | 6 |  |
| Household wealth index ^a^ | |  |  | <0.001 |
|  | Lowest | 33 (28.2) | 26 (12.4) |  |
|  | Second | 15 (12.8) | 47 (22.4) |  |
|  | Middle | 15 (12.8) | 57 (27.1) |  |
|  | Fourth | 18 (15.4) | 51 (24.3) |  |
|  | Highest | 36 (30.8) | 29 (13.8) |  |
| Type of admission | |  |  | 0.921 |
|  | New admission | 110 (94.0) | 198 (94.3) |  |
|  | Re-admission/Transfer from ITC | 7 (6.0) | 12 (5.7) |  |
| Poor appetite | | 34 (29.1) | 44 (30.0) | 0.100 |
| Cough | | 28 (23.9) | 38 (18.1) | 0.208 |
| Vomit | | 10 (8.6) | 14 (6.7) | 0.532 |
| Diarrhoea | | 23 (19.7) | 29 (13.8) | 0.166 |
| Fever | | 35 (29.9) | 34 (16.2) | 0.004 |
| Skin abnormality | | 8 (6.8) | 13 (6.2) | 0.819 |

**Supplemental Table S1: Characteristics of study participants after excluding children enrolled in the study at the same time as children with missing outcome data.**

Data presented as n (%) for categorical variables or Mean ±SD for continuous variables. ^a^ Derived using principal components analysis of household assets, access to utilities and type of housing material.

| **Outcome** | **Control**  (N=117) |  | **Intervention** (N=210) |
| --- | --- | --- | --- |
|  | n (%) |  | n (%) |
| Cured | 89 (76.1) |  | 190 (90.5) |
| Defaulted | 24 (20.5) |  | 13 (6.5) |
| Transferred to ITC | 3 (2.6) |  | 3 (1.4) |
| Died | 1(0.9) |  | 2 (1.0) |
| No response | 0 (0.0) |  | 2 (1.0) |
| Length of stay (days)^a^ | 32.2±19.2 |  | 34.3±18.2 |
| Average weight gain (g/kg/day)^a^ | 6.6±4.6 |  | 6.3±3.9 |

**Supplemental Table S2: Treatment outcomes after excluding children enrolled in the study at the same time as children with missing outcome data.**

^a^ Applies to cured children only. Data presented as Mean ±SD

| **Outcome** | **Unadjusted** | | | |  | **Adjusted^1^** | | | |
| --- | --- | --- | --- | --- | --- | --- | --- | --- | --- |
|  | **RR (95% CI)** | **P value** | **RD or MD (95% CI)** | **P value** |  | **RR (95% CI)** | **P value** | **RD or MD (95% CI)** | **P value** |
| Cure (N=327) | 1.19 (1.06, 1.33) | 0.002 | 0.14* (0.06, 0.23) | 0.001 |  | 1.15 (1.02, 1.29) | 0.022 | 0.12* (0.02, 0.21) | 0.017 |
| Default (N=327) | 0.30 (0.16, 0.57) | <0.001 | -0.14* (-0.22, -0.06) | <0.001 |  | 0.32 (0.16, 0.64) | 0.001 | -0.14* (-0.23, -0.06) | 0.001 |
| Length of stay (N=279) | -- |  | 2.07^†^ (-2.62, 6.77) | 0.385 |  | -- |  | 3.70^†^ (-1.30, 8.69) | 0.146 |
| Average weight gain (N=279) | -- |  | -0.23^†^ (-1.29, 0.83) | 0.676 |  | -- |  | -0.81^†^ (-1.95, 0.33) | 0.165 |

**Supplemental Table S3: Effect of the intervention on treatment outcomes after excluding children enrolled in the study at the same time as children with missing outcome data.**

^1^Adjusted for wealth index, exposure to HIV, and fever

* Risk difference

^†^Mean difference

RR: Risk ratio; RD: Risk difference; MD: Mean Difference

The effects of the intervention on transfer, death and no response were not evaluated because of the small number of cases
